# The Gene Expression Biomarkers for Chronic Obstructive Pulmonary Disease and Interstitial Lung Disease

**DOI:** 10.3389/fgene.2019.01154

**Published:** 2019-11-20

**Authors:** Yangwei Yao, Yangyang Gu, Meng Yang, Dakui Cao, Fengjie Wu

**Affiliations:** Department of Pulmonary and Critical Care Medicine, The Second Hospital of Jiaxing, Jiaxing, China

**Keywords:** chronic obstructive pulmonary disease, interstitial lung disease, biomarker, gene expression, treatment target

## Abstract

COPD (chronic obstructive pulmonary disease) and ILD (interstitial lung disease) are two common respiratory diseases. They share similar clinical traits but require different therapeutic treatments. Identifying the biomarkers that are differentially expressed between them will not only help the diagnosis of COPD and ILD, but also provide candidate drug targets that may facilitate the development of new treatment for COPD and ILD. Due to the irreversible complex pathological changes of COPD, there are very limited therapeutic options for COPD patients. In this study, we analyzed the gene expression profiles of two datasets: one training dataset that includes 144 COPD patients and 194 ILD patients, and one test dataset that includes 75 COPD patients and 61 ILD patients. Advanced feature selection methods, mRMR (minimal Redundancy Maximal Relevance) and incremental feature selection (IFS), were applied to identify the 38-gene biomarker. An SVM (support vector machine) classifier was built based on the 38-gene biomarker. Its accuracy, sensitivity, and specificity on training dataset evaluated by leave one out cross-validation were 0.905, 0.896, and 0.912, respectively. And on independent test dataset, the accuracy, sensitivity, and specificity on were as great as and were 0.904, 0.933, and 0.869, respectively. The biological function analysis of the 38 genes indicated that many of them can be potential treatment targets that may benefit COPD and ILD patients.

## Introduction

COPD (chronic obstructive pulmonary disease) and ILD (interstitial lung disease) are both common lung diseases (Andersen et al., 2013). And cigarette smoking is the biggest risk factor for COPD and ILD (Caminati et al., 2012). About 20% smokers will develop COPD (Bosse, 2012). COPD is also an independent risk factor of lung cancer. Both emphysema and non-emphysema COPD phenotypes significantly increased the risk of lung cancer (Wang et al., 2018). In addition, epidemiological studies have found that COPD increases the risk of lung cancer by two to six times, regardless of whether there is a history of smoking or not (Papi et al., 2004; Young et al., 2009). Since the complex pathological changes in COPD and most of ILD patients are not irreversible, the diseases cause extensive mortality and are great public health problems worldwide (Vogelmeier et al., 2017).

Although COPD and ILD share many common traits and have similar clinical phenotypes, their treatments and the therapeutic effects are different. The recommended treatments for COPD patients are smoking cessation and drugs that treat bronchoconstriction and inflammation, such as methylxanthines, β-adrenoceptor agonists, corticosteroids, phosphodiesterase type 4 (PDE-4) inhibitors, and anticholinergics (Andersen et al., 2013), while the ILD patients are treated with immunosuppressive agents, such as alkylating nitrogen mustard (du Bois, 2010). Inhaled corticosteroids (ICS) are important in managing exacerbations and symptoms in COPD (Lakshmi et al., 2017). However, a significant percentage of patients respond poorly or not at all to pharmacotherapies, especially for patients with severe disease (Nixon et al., 2017). In addition, poor adherence to medication is an essential factor in treatment failure. Therefore, new therapy strategies are needed urgently.

It is critical to classify COPD patients from ILD patients since it is the first step for choosing the right treatment. As we mentioned, COPD and ILD share similar pathogeny and have similar clinical phenotype; it is difficult to discriminate these two diseases and the underlying mechanisms of COPD and ILD are largely unknown. Identifying the biomarkers for COPD and ILD will not only provide a tool for disease diagnosis, but also reveal novel insights of the pathological mechanisms and help developing new treatment to benefit the survival of patients. Microarray is a reliable technology to measure the expression level of thousands of genes simultaneously and has been proven to be great data source for discovering biomarkers.

In this study, we analyzed two gene expression datasets of COPD and ILD: one training dataset of Agilent-028004 SurePrint G3 Human GE 8x60K Microarray including 144 COPD patients and 194 ILD patients, and one independent test data of Agilent-014850 Whole Human Genome Microarray 4x44K G4112F including 75 COPD patients and 61 ILD patients. Advanced feature selection methods, mRMR (minimal Redundancy Maximal Relevance) and IFS (incremental feature selection), were applied to get the optimal biomarkers on training dataset. The SVM (support vector machine) method was used to construct the classifier on training dataset and tested on independent test dataset. The 37-gene classifier achieved great performance on training and test datasets. The accuracies on training data and test data were 0.964 and 0.904, respectively. The 37 selected genes were involved in key biological pathways and functions of COPD and ILD. These results provided novel insight for understanding the mechanisms of COPD and ILD and shed light on new treatment development.

## Methods

### The Gene Expression Profiles of COPD and ILD Patients

The gene expression profiles of COPD and ILD patients were downloaded from GEO (Gene Expression Omnibus) with accession number of GSE47460 (https://www.ncbi.nlm.nih.gov/geo/query/acc.cgi?acc=GSE47460). The original data were generated by Peng et al. (2016). They measured the gene expression levels of 144 COPD patients and 194 ILD patients with Agilent-028004 SurePrint G3 Human GE 8x60K Microarray and 75 COPD patients and 61 ILD patients with Agilent-014850 Whole Human Genome Microarray 4x44K G4112F. We extracted the common 15,180 genes between these two microarray platforms and quantile normalized the two datasets. Then the first dataset of 144 COPD patients and 194 ILD patients were considered as training dataset, while the second dataset of 75 COPD patients and 61 ILD patients were considered as independent test dataset.

### Biomarker Selection Using mRMR and IFS Methods

We adopted the mRMR (minimal Redundancy Maximal Relevance) method (Peng et al., 2005) to rank the genes on the training dataset. The mutual information-based mRMR method is widely used and has been used in solving many biomedical problems (Niu et al., 2013; Zhao et al., 2013; Zhou et al., 2015). The C/C++ version mRMR program was downloaded from http://home.penglab.com/proj/mRMR/. Unlike the univariate method, such as t test and ANOVA (analysis of variance), mRMR considers not only the relevance between genes and disease types but also the redundancies between genes.

?, Ω*_s_*, and Ω*_t_* were used to represent the complete set of all 15,180 (N) candidate genes for biomarker ranking, the selected m genes, and the to-be-selected n genes, respectively. The relevance of gene g from Ω*_t_* with disease type t can be measured with mutual information (*I*) (Sun et al., 2012; Huang and Cai, 2013):

(1) D=I(g,t)

And the redundancy R of the gene g with the selected genes in Ω*_s_* are

(2) $$\text{R}=\frac{1}{m}\left(\sum_{g_{i}\in \Omega_{s}}I\left(g,g_{i}\right)\right)$$

The goal of this algorithm is to get the gene *g* *_j_* from Ω*_s_* that has maximum relevance with disease type t and minimum redundancy with the selected genes in Ω*_s_*, i.e. maximize the mRMR function

(3) $$\underset{g_{j}\in \Omega_{t}}{\text{max}}\left[I\left(g_{j},t\right)-\frac{1}{m}\left(\sum_{g_{i}\in \Omega_{s}}I\left(g,g_{i}\right)\right)\;\;\right]\;\left(j=1,2,\ldots ,n\right)\;\;\;\;\;$$

The evaluation procedure will be continued for N rounds, and all the genes will be ranked as a list

(4) $$\text{S}=\left\{g_{1}^{'},g_{2}^{'},\ldots ,g_{h}^{'},\ldots ,g_{N}^{'}\right\}$$

The index h reflects the trade-off between relevance with disease type and redundancy with selected genes. The smaller the index h is, the better the discriminating power the gene has.

Based on the top 500 mRMR genes, we constructed 500 SVM classifiers and applied an IFS method (Jiang et al., 2013; Li et al., 2014; Shu et al., 2014; Zhang et al., 2014a; Zhang et al., 2015) to identify the optimal genes as biomarker. Each candidate gene set $S_{k}=\left\{g_{1}^{'},g_{2}^{'},\ldots ,g_{k}^{'}\right\}\left(1\leq k\leq 500\right)$ included the top k genes in the mRMR list.

Based on the leave-one-out cross-validation (LOOCV) accuracy of each candidate gene set on the training dataset, an IFS curve can be plotted. The x-axis denoted the number of top genes that were used to train the SVM classifier, and the y-axis denoted the LOOCV accuracies of trained classifiers. Based on the IFS curve, we can choose the right cutoff of gene numbers to achieve a good prediction performance.

### Prediction Performance Evaluation of the Classifier

**Figure 1:**
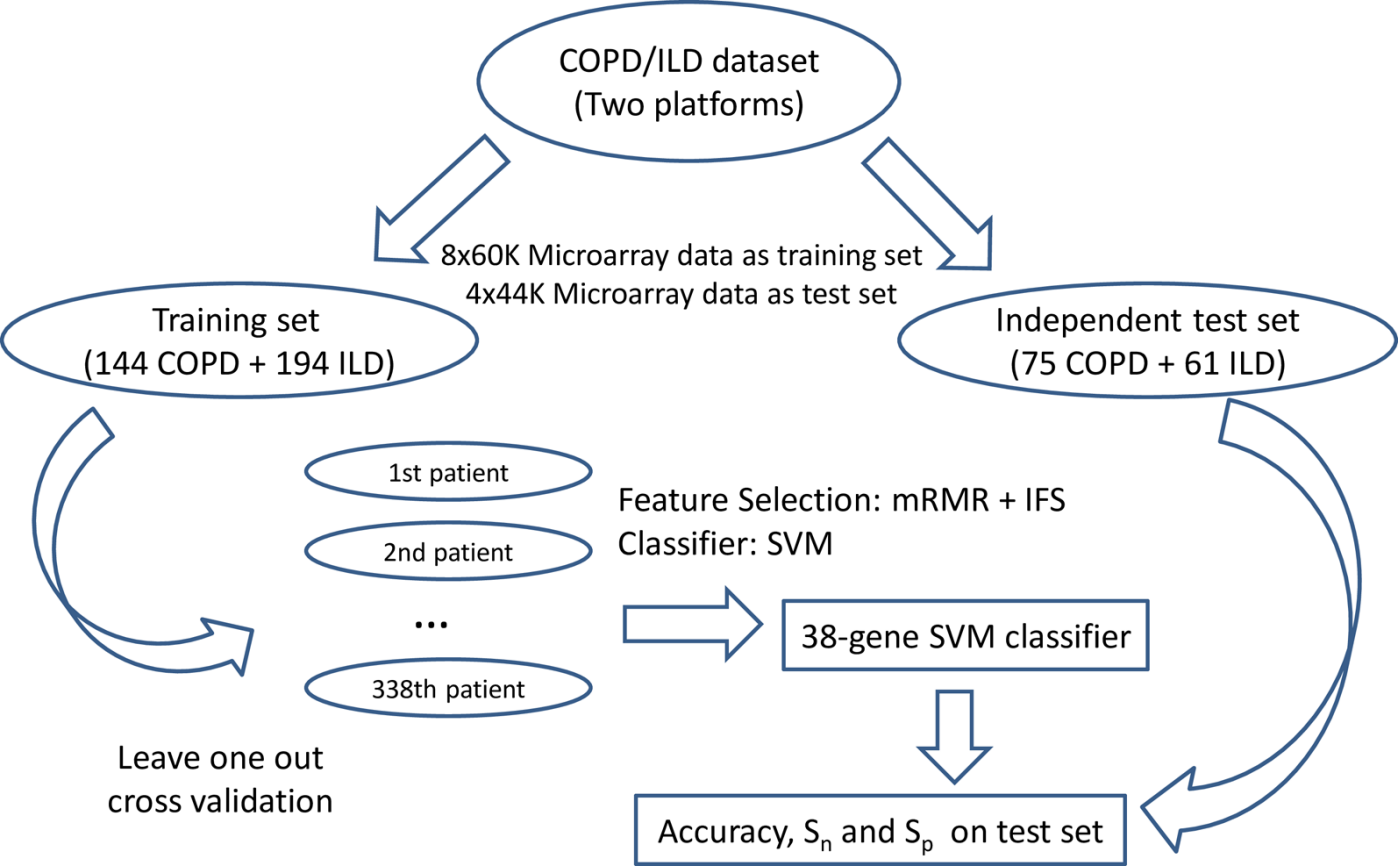
The flowchart of biomarker selection, classifier construction, and prediction performance evaluation. First, the COPD/ILD samples were divided into training dataset and test dataset based on their platform: the 144 COPD samples and 194 ILD samples profiled with 8x60K Microarray was the training set; the 75 COPD samples and 61 ILD samples profiled with 4x44K Microarray were the test set. Then in the training set, we applied mRMR and IFS to select the optimal number of genes as biomarkers and evaluated its performance on the training dataset using leave-one-out cross-validation. At last, the final 38-gene SVM classifier was trained using all training dataset and tested on the independent test dataset. The accuracy, sensitivity, and specificity were calculated to objectively evaluate the prediction performance of the 38-gene classifier.

We used LOOCV (Cui et al., 2013; Yang et al., 2014) to evaluate the prediction performance of the SVM classifiers on the training dataset and then independently tested the final classifier that was trained using all training data on the independent test dataset. During LOOCV on training dataset, all of the N training samples were tested one by one. In each round, one sample was used for testing of the prediction model trained with all the other N-1 samples. After N rounds, all samples were tested one time, and the predicted disease types were compared with the actual disease types. The final classifier was trained using all the training samples and then tested on the independent test dataset. **Figure 1** showed the flowchart of biomarker selection, classifier construction, and prediction performance evaluation. The SVM method was applied using the svm function with default parameters in R package e10171 (https://cran.r-project.org/web/packages/e1071/).

Accuracy (ACC), Sensitivity (Sn), and Specificity (Sp) were calculated to evaluate the prediction performance

(5) $$\;ACC=\frac{TP+TN}{TP+TN+FP+FN}\;$$

(6) $$S_{n}=\frac{TP}{TP+FN}\;$$

(7) $$S_{p}=\frac{TN}{TN+FP}$$

where TP, TN, FP, and FN stand for true positive (COPD), true negative (ILD), false positive (COPD), and false negative (ILD), respectively.

## Results and Discussion

### The genes that showed different expression pattern between COPD and ILD patients

We obtained the top 500 most discriminative genes of COPD and ILD patient samples using the mRMR method on the training dataset. The mRMR ranked the genes based on their relevance with disease types, COPD or ILD, and their redundancy with selected genes. Both the relevance and redundancy were measured with mutual information. The mutual information has been proven to be a better statistic than correlation and was widely used. The top 500 mRMR genes were given in **Table S1**.

### The Optimal Biomarkers Identified From the mRMR Gene List With IFS Methods

**Figure 2:**
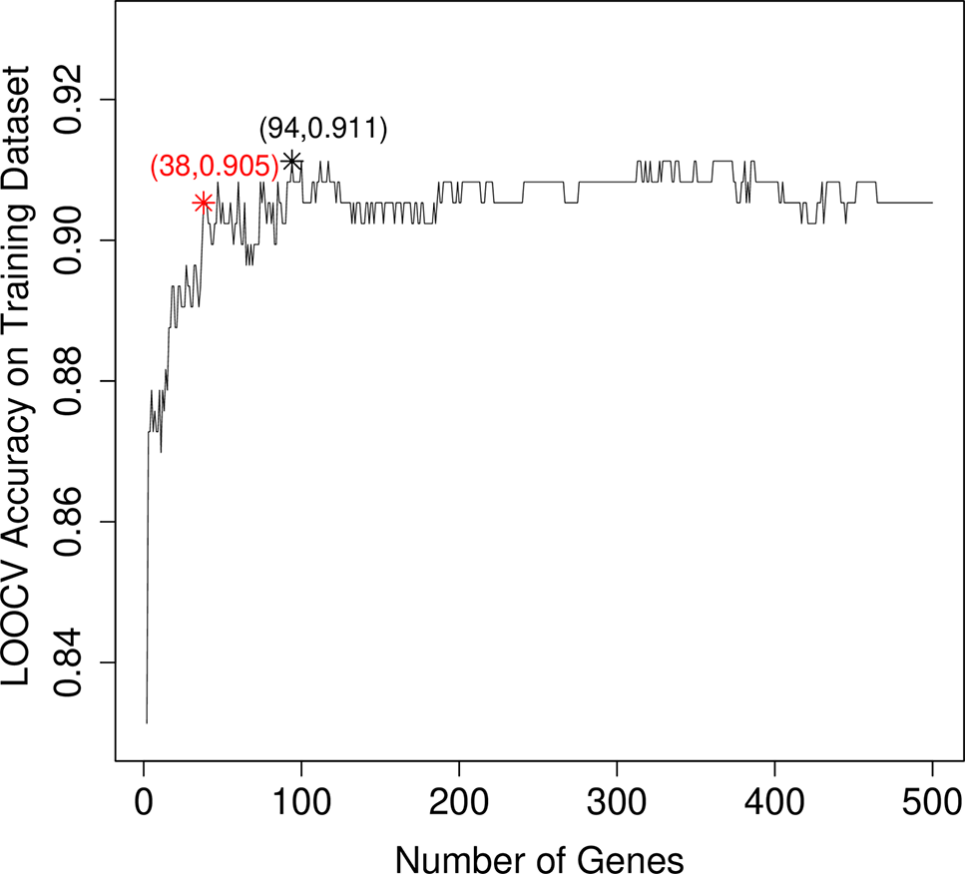
The IFS curve that showed how the prediction performance improved when more and more genes were used to construct the classifier. The IFS curve explained the relationship between the number of genes and prediction accuracies. The x-axis denoted the number of top genes that were used to train the SVM classifier, and the y-axis denoted the LOOCV accuracies of trained classifiers. The highest accuracy was achieved when 94 genes were used. But after 38 genes were used, the IFS curve entered the plateau area and did not increase too much even when more and more genes were included. To consider both the model complexity and model performance, we chose the 38 genes as the optimal biomarker gene set.

After mRMR analysis, the genes were ranked based on the gene expression profiles on training dataset. But we still did not know how many top genes should we choose. And the ideal biomarkers should use less genes and achieve great performance. Therefore, we applied the IFS procedure to select the optimal number of top mRMR genes to form the biomarker gene set. During each round of IFS, different numbers of top genes were used and the corresponding prediction performance, i.e., the LOOCV accuracy on training dataset, were calculated. The relationship between the number of genes and prediction accuracies was plotted as an IFS curve as shown in **Figure 2**. It can be seen that when 94 genes were used, the LOOCV accuracy on training dataset was the highest. But even early, when only 38 genes were used, the accuracy was over 0.90. To consider both using less genes and achieving higher prediction accuracy, we chose the 38 genes as the optimal biomarker gene set since increasing the number of genes will not significantly increase the accuracy any more after the 38 genes were used. The 38 genes were shown in **Table 1**.

**Table 1:** The 38 genes selected by mRMR and IFS methods.

Order	Symbol	Name	Score
1	HBEGF	Heparin binding EGF like growth factor	0.288
2	DIO2	Iodothyronine deiodinase 2	0.187
3	CLCN3	Chloride voltage-gated channel 3	0.115
4	SEPT4	Septin 4	0.120
5	FAT1	FAT atypical cadherin 1	0.120
6	CTSE	Cathepsin E	0.116
7	CRIP1	Cysteine rich protein 1	0.108
8	ACADVL	Acyl-CoA dehydrogenase, very long chain	0.112
9	CNTN3	Contactin 3	0.118
10	UQCRQ	Ubiquinol-cytochrome c reductase complex III subunit VII	0.116
11	ASPN	Asporin	0.111
12	ZNF786	Zinc finger protein 786	0.110
13	RARRES2	Retinoic acid receptor responder 2	0.107
14	BTC	Betacellulin	0.111
15	FNDC1	Fibronectin type III domain containing 1	0.114
16	DUSP1	Dual specificity phosphatase 1	0.113
17	C6orf145	PX domain containing 1	0.104
18	NUTF2	Nuclear transport factor 2	0.105
19	TNN	Tenascin N	0.101
20	COQ9	Coenzyme Q9	0.103
21	SCG5	Secretogranin V	0.105
22	BCHE	Butyrylcholinesterase	0.099
23	NR4A2	Nuclear transport factor 2	0.100
24	HS6ST3	Heparan sulfate 6-O-sulfotransferase 3	0.103
25	SHE	Src homology 2 domain containing E	0.102
26	C20orf111	Oxidative stress responsive serine rich 1	0.098
27	REEP2	Receptor accessory protein 2	0.099
28	C19orf63	ER membrane protein complex subunit 10	0.097
29	IRS2	Nuclear receptor subfamily 4 group A member 2	0.098
30	FA2H	Fatty acid 2-hydroxylase	0.094
31	ACTL6A	Actin like 6A	0.094
32	NR4A3	Nuclear receptor subfamily 4 group A member 3	0.093
33	DAO	D-amino acid oxidase	0.095
34	VNN2	Vanin 2	0.093
35	IGFL2	IGF like family member 2	0.094
36	ZNF692	Zinc finger protein 692	0.093
37	CAMK1D	Calcium/calmodulin-dependent protein kinase ID	0.091
38	HCAR2	Hydroxycarboxylic acid receptor 2	0.092

### The Prediction Performance of the 38-Gene Classifier

The 38 genes were chosen from the genome wide 15,180 genes based on mRMR and IFS methods. To objectively evaluate their prediction power, we calculated not only the LOOCV accuracy, sensitivity, and specificity on training dataset, but also the accuracy sensitivity and specificity on independent test dataset. The confusion matrix of predicted disease types and actual disease types on both training and test datasets were shown in **Table 2**. On training dataset, the LOOCV accuracy, sensitivity, and specificity were 0.905, 0.896, and 0.912, respectively. More importantly, the accuracy, sensitivity, and specificity on independent test dataset were as great as on the training dataset and were 0.904, 0.933, and 0.869, respectively.

**Table 2:** The confusion matrix of predicted disease types and actual disease types on both training and test datasets.

Leave one out cross validation on Training set*			Independent test on test set*		
	Actual COPD	Actual ILD		Actual COPD	Actual ILD
Predicted COPD	129	17	Predicted COPD	70	8
Predicted ILD	15	177	Predicted ILD	5	53
Accuracy: 0.905	Sensitivity: 0.896	Specificity: 0.912	Accuracy: 0.904	Sensitivity: 0.933	Specificity: 0.869
*COPD was considered as positive sample and ILD was considered as negative samples during sensitivity and specificity calculation.					

**Figure 3:**
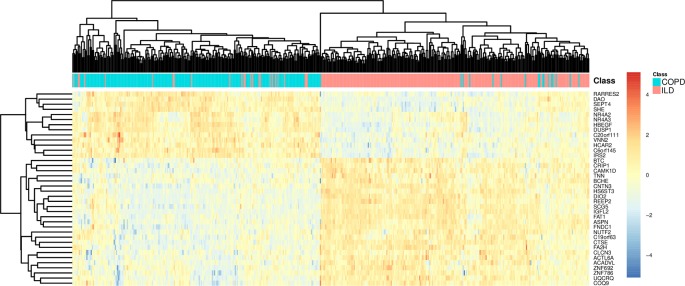
The heatmap of COPD and ILD patients using the selected 38 genes. The COPD and ILD patients from training dataset and test dataset were hierarchically cluttered using the 38 selected genes. There were very clear clusters of COPD and cluster of ILD. Most samples were grouped into the right cluster.

To more intuitively demonstrate the discriminative power of these 38 genes for COPD and ILD samples, we combined the training dataset samples and test dataset samples and draw a heatmap using these 38 genes (**Figure 3**). It can be seen that even without advanced machine learning algorithm, such as SVM, the simple hierarchical clustering can group most COPD and ILD samples into the right clusters. And the upregulation and downregulation patterns of these 38 genes were very clear between COPD and ILD patients.

We also calculated the results of the 94 genes and plotted their heatmap as **Figure S1**. On training dataset, the LOOCV accuracy, sensitivity, and specificity of the 94-gene classifier were 0.911, 0.889, and 0.928, respectively. On independent test dataset, the accuracy, sensitivity, and specificity of the 94-gene classifier were 0.897, 0.933, and 0.852, respectively. The performance of the 94 genes was close to the 38 genes on both training and independent test datasets. The 38 genes were even slightly better than the 94 genes on independent test dataset.

### The Biological Significance of the 38-Gene Biomarkers

**Figure 4:**
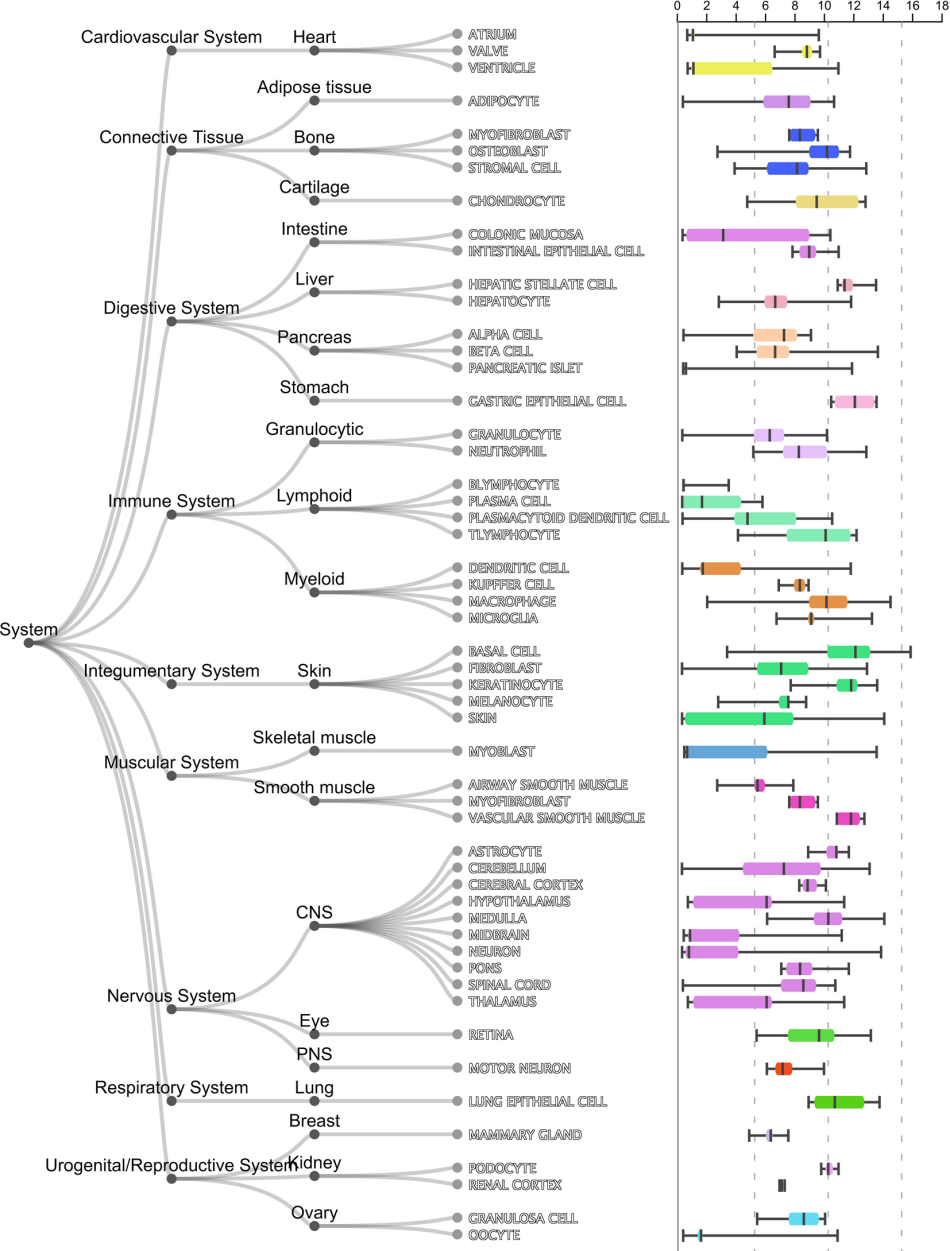
The tissue specific expression pattern of HBEGF in ARCHS4. The tissue expression data from ARCHS4 showed that HBEGF is very specifically highly expressed in lung (https://amp.pharm.mssm.edu/archs4/gene/HBEGF#tissueexpression).

As shown in **Table 1**, the first gene on the mRMR list was HBEGF (heparin binding EGF like growth factor). From **Figure 2**, it can be seen that HBEGF was highly expressed in COPD patients. HBEGF is a key member of the EGFR pathway. Its expression level has been reported to be increased in COPD samples and were significantly correlated with both diffusing capacity of the lung for carbon monoxide (DLCO) and Forced Expiratory Volume in 1 second (FEV1), two major clinical variables for COPD (Cockayne et al., 2012). We investigated the tissue specific expression pattern of HBEGF in ARCHS4 (Lachmann et al., 2018) and **Figure 4**, which were retrieved from ARCHS4, showed that HBEGF is very specifically highly expressed in lung.

The second gene was DIO2 (iodothyronine deiodinase 2). DIO2 plays an important role in biologically active triiodothyronine synthesis. Its expression level was consistent with the degree of lung injury: the more the lung injury, the higher the expression of DIO2 (Ma et al., 2011). Clearly, DIO2 is key for the inflammatory response (Ma et al., 2011). And COPD is a complex chronic inflammatory disease involving the dysfunction of a variety of inflammatory mediators (Thorley and Tetley, 2007). DIO2 could be a key factor in the inflammatory mechanism of COPD (Barnes, 2017).

CLCN3 (chloride voltage-gated channel 3) ranked third on the mRMR list. It has been reported that the CLCN3 mRNA was expressed in fetal airway epithelia and played important roles in pulmonary epithelium developing of human lung (Lamb et al., 2001). As we have known, COPD mainly affects pulmonary epithelium (Hiemstra et al., 1998). And it is believed that cigarette smoke triggers COPD through causing epithelial damage and interfering repair processes (Thorley and Tetley, 2007).

ILD and COPD are two kinds of chronic lung diseases with significant differences in etiology, incidence, pathology, and prognosis (McDonald, 2018). ILD is a heterogeneous group of diseases, characterized by chronic, progressive, mainly interstitial inflammation and is always accompanied by varying degrees of pulmonary parenchyma fibrosis (Doyle et al., 2012), while COPD is characterized by chronic airflow limitation caused by small airway disease and substantial destruction, which is not completely reversible and usually progressive (Song et al., 2012; Rabe and Watz, 2017). Generally, the diagnosis and classiﬁcation of ILD and COPD severity depend on clinical evaluation, thoracic imaging, and pulmonary function testing (PFT) (Song et al., 2012; Du Plessis et al., 2018).

Among these identified genes, HBEGF has been found related with the invasion and progression of many malignant tumors including breast, pancreatic, and ovarian, and may be involved in macrophage-mediated cellular proliferation (Ray et al., 2014; He et al., 2015). He et al. (2019) conducted comprehensive bioinformatic analyses to predict target genes of ILD and identified HBEGF as one of the potential prognostic markers and therapeutic targets for ILD. Besides, SEPTIN4, a member of the septin family of nucleotide binding proteins, plays a role in apoptosis and cancer (Garcia et al., 2008), which may affect the occurrence and development of ILD.

We will not go through the mRMR table one by one. With only the top three genes, the LOOCV accuracy was 0.873 as shown in **Figure 2**. There are several genes in **Table 1** that are highly possible to play key roles in COPD. Notably, CTSE (cathepsin E) ranked sixth was reported to be able to promote pulmonary emphysema through causing mitochondrial fission and may be a candidate therapeutic target (Zhang et al., 2014b). BTC (betacellulin) ranked 14th was found to be higher expressed in COPD ex-smokers than ex-smokers without COPD (de Boer et al., 2006). DUSP1 (dual specificity phosphatase 1) ranked 16th was reported to have anti-inflammatory potential (Newton, 2014) and when COPD patients undertook fluticasone propionate, DUSP1 expression level was increased (Lee et al., 2016). BCHE (butyrylcholinesterase) ranked 22nd was associated with oxidative stress and inflammation, and its activity was found to be decreased in COPD patients (Sicinska et al., 2017). In **Figure 3**, we also observed the downregulation of BCHE in COPD cluster. SHE (Src homology 2 domain containing E) ranked 25th may play a critical role in promoting airway smooth muscle cell growth and migration during the airway remodeling of COPD patients (Krymskaya et al., 2005). DAO (D-amino acid oxidase) ranked 33rd was an enzyme for peroxisome, glyoxylate metabolism, and glycine degradation. The serum DAO activity was found to be higher in the intestinal tissue of COPD model rat than control (Xin et al., 2016). CAMK1D (calcium/calmodulin dependent protein kinase ID) ranked 37th was found to be a hub node on the protein–protein interaction network of differentially expressed gene (DEG) in COPD and was considered as candidate biomarker and potential target for clinical diagnosis and treatment of COPD (Yuan et al., 2014).

Since there are very few drugs for COPD, we searched DrugBank for possible COPD drugs and found that BCHE, DAO, UQCRQ, HCAR2, CAMK1D, and NR4A3 were drug targetable. The number of small molecule drugs that targeted BCHE, DAO, UQCRQ, HCAR2, CAMK1D, and NR4A3 were 31, 8, 8, 3, 2, and 1, respectively. These genes can be considered as therapeutic targets and may be helpful for development of COPD treatment.

### The Associations Between the 38 Genes and Air Pollutants, Particulate Matter, and Tobacco Smoke Pollution

COPD has a close relationship with environmental factors. Pollution and smoking can trigger COPD. Some of the 38 genes have been reported to be associated with smoking by GWAS (genome-wide association study). For example, rs1374879 within CNTN3, which ranked 9th in **Table 1**, was found to be associated with smoking quantity (Argos et al., 2014). Therefore, we systematically studied the associations between signature genes and air pollutants, particulate matter, and tobacco smoke pollution in CTD (comparative toxicogenomics database) (Mattingly et al., 2006). **Table 3** listed how many manually curated literatures, the associations between the gene, and the environmental factor were reported.

**Table 3:** The associations between the 38 genes and air pollutants, particulate matter, and tobacco smoke pollution.

Gene	Air pollutants*	Particulate matter*	Tobacco smoke pollution*
HBEGF	1	15	5
DIO2	0	5	1
CLCN3	0	0	0
SEPT4	0	1	1
FAT1	0	3	2
CTSE	0	4	4
CRIP1	1	1	0
ACADVL	0	4	0
CNTN3	0	0	0
UQCRQ	0	0	0
ASPN	0	0	0
ZNF786	0	0	0
RARRES2	0	3	1
BTC	0	0	0
FNDC1	0	2	1
DUSP1	1	12	3
C6orf145	0	0	0
NUTF2	0	1	1
TNN	0	2	1
COQ9	0	0	0
SCG5	0	1	0
BCHE	0	2	0
NR4A2	1	3	1
HS6ST3	0	0	0
SHE	0	0	0
C20orf111	0	0	0
REEP2	0	0	0
C19orf63	0	0	0
IRS2	0	2	1
FA2H	0	1	0
ACTL6A	1	1	0
NR4A3	1	2	1
DAO	0	1	1
VNN2	1	1	1
IGFL2	0	0	0
ZNF692	0	0	0
CAMK1D	0	3	1
HCAR2	0	2	1
*: The number literatures that suggested the association.			

It can be seen that 5 genes (HBEGF, DUSP1, NR4A2, NR4A3, and VNN2) were associated with all three environmental factors, 14 genes were associated with two environmental factors, and 4 genes were associated with one environmental factor. Column wise, there were 23 genes associated with particulate matter, 17 genes associated with tobacco smoke pollution, and 7 genes associated with air pollutants. Particulate matter is a serious threat to health and can cause many lung diseases (Shu et al., 2016).

## Conclusion

COPD and ILD are two common and similar lung diseases. Both diseases cause huge public health problems. The diagnosis of COPD and ILD is essential for early treatment. We analyzed the gene expression profiles of COPD and ILD patients from two batches that were measured with two microarray platforms. We chose one dataset as the training dataset and selected 38 genes that showed different expression pattern between COPD and ILD patients using advanced mRMR and IFS methods. Based on these 38 genes, a powerful COPD/ILD SVM classifier was built. The classifier had great performance both on training dataset evaluated by LOOCV and on independent test dataset. The 38-gene classifier demonstrated great robustness and excellent prediction accuracy. The biological function analysis of the 38 genes indicated that many of them can be potential treatment targets that may improve the current COPD and ILD therapeutic practice.

## Data Availability Statement

All datasets generated for this study are included in the article/**Supplementary Material**.

## Author Contributions

YY contributed to the study design. YG conducted the literature search. MY acquired the data. DC wrote the article. FW performed data analysis. YY revised the article and gave the final approval of the version to be submitted. All authors read and approved the final manuscript.

## Funding

This study was supported by the funds from the Funds of Science and Technology Project of Jiaxing (2017AY33029).

## Conflict of Interest

The authors declare that the research was conducted in the absence of any commercial or financial relationships that could be construed as a potential conflict of interest.

## Supplementary Material

Table S1 The top 500 mRMR gene that showed different expression pattern between COPD and ILD patients. The first column is the rank and the second column is the gene symbol.

Figure S1 The heatmap of COPD and ILD patients using the top 94 genes. The COPD and ILD patients from training dataset and test dataset were hierarchically cluttered using the top 94 genes. There were very clear cluster of COPD and cluster of ILD. Most samples were grouped into the right cluster.
