# Supplementary figures and images for: The Gene Expression Biomarkers for Chronic Obstructive Pulmonary Disease and Interstitial Lung Disease
Source: Front Genet. 2019 Nov 20;10:1154. doi: 10.3389/fgene.2019.01154 (PMC6879656; doi:10.3389/fgene.2019.01154)

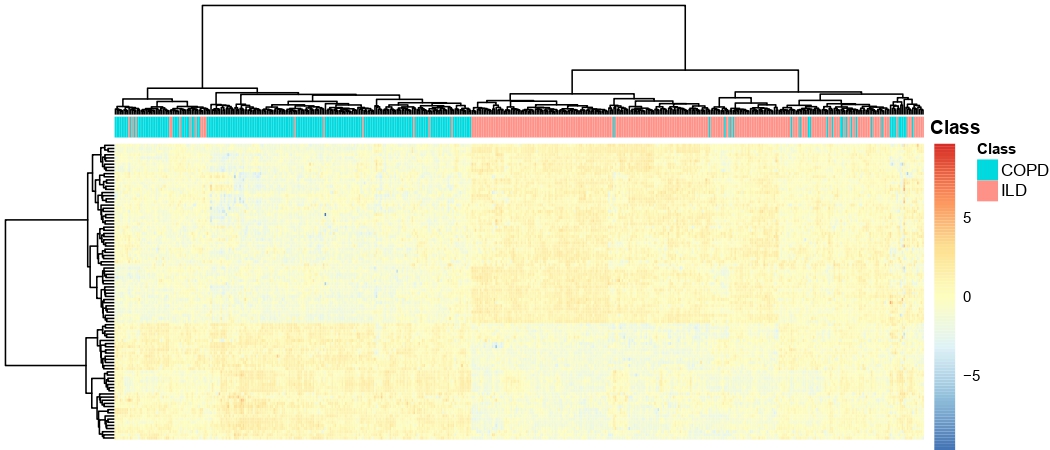

Supplement: Figure S1 — The heatmap of COPD and ILD patients using the top 94 genes. The COPD and ILD patients from training dataset and test dataset were hierarchically cluttered using the top 94 genes. There were very clear cluster of COPD and cluster of ILD. Most samples were grouped into the right cluster. [file Image_1.jpeg]
